# A meta‐analysis of the relationship between *VEGFR2* polymorphisms and atherosclerotic cardiovascular diseases

**DOI:** 10.1002/clc.23233

**Published:** 2019-07-24

**Authors:** Li Wang, Hui Ge, Longyun Peng, Bing Wang

**Affiliations:** ^1^ Department of Healthcare First Affiliated Hospital, Sun Yat‐sen University Guangzhou China; ^2^ Department of Cardiology First Affiliated Hospital, Sun Yat‐sen University Guangzhou China; ^3^ Department of Radiology Second Affiliated Hospital, Army Medical University Chongqing China

**Keywords:** atherosclerotic cardiovascular diseases, coronary artery disease, ischemic stroke, meta‐analysis, polymorphisms, vascular endothelial growth factor receptor 2

## Abstract

**Background:**

Some previous studies explored associations between vascular endothelial growth factor receptor 2 (*VEGFR2*) polymorphisms and atherosclerotic cardiovascular diseases (ASCVD), with conflicting findings.

**Hypothesis:**

We thought that *VEGFR2* polymorphisms may influence susceptibility to ASCVD. Here, we aimed to better analyze the relationship between *VEGFR2* polymorphisms and ASCVD in a larger combined population by performing a meta‐analysis.

**Methods:**

We searched Pubmed, Embase, and Web of Science for related articles. We calculated odds ratio (OR) and 95% confidence interval (CI) to estimate whether there are genetic associations between *VEGFR2* polymorphisms and ASCVD**.**

**Results:**

Ten studies were included for this meta‐analysis (5474 cases and 8584 controls). *VEGFR2* rs1870377 (dominant comparison: 0.81 (0.73‐0.89), *I* ^2^ = 56%; recessive comparison: 1.40 (1.25‐1.57), *I* ^2^ = 34%; allele comparison: 0.81 (0.76‐0.87), *I* ^2^ = 0%), rs2071559 (dominant comparison: 0.83 (0.76‐0.91), *I* ^2^ = 0%; recessive comparison: 1.22 (1.07‐1.38), *I* ^2^ = 0%; allele comparison: 0.86 (0.81‐0.92), *I* ^2^ = 0%) and rs2305948 (dominant comparison: 0.79 (0.72‐0.87), *I* ^2^ = 25%; recessive comparison: 1.44 (1.08‐1.92), *I* ^2^ = 60%; allele comparison: 0.79 (0.68‐0.92), *I* ^2^ = 73%) polymorphisms were all found to be significantly associated with susceptibility to ASCVD in general population. Subgroup analyses by type of disease revealed similar significant findings for rs1870377, rs2071559, and rs2305948 polymorphisms in coronary artery disease (CAD) subgroup. Besides, positive results were also found for rs1870377 polymorphism in ischemic stroke (IS) subgroup.

**Conclusions:**

In summary, this meta‐analysis proved that these *VEGFR2* polymorphisms could be used to identify individual with elevated susceptibility to ASCVD.

## 1 INTRODUCTION

Atherosclerotic cardiovascular diseases (ASCVD) usually manifest as coronary artery disease (CAD), ischemic stroke (IS) and peripheral arterial disease (PAD).^1^ It poses a huge threat to public health and is the leading cause of death all over the world.^2^ Although the precise pathogenesis mechanism of ASCVD is still unrevealed, it was thought that genetic factors may contribute a lot to its development. First, the prevalence of ASCVD varies greatly across different populations,^3^ and difference in genetic components is likely to be one of the reasons for this variation in disease prevalence. Second, previous genetic association studies showed that many genetic loci were significantly associated with an increased susceptibility to ASCVD.^4^, ^5^, ^6^ Moreover, using the combination of these susceptible genetic loci to predict the risk of developing ASCVD in general population was also demonstrated to be effective and cost‐saving.^7^

Vascular endothelial growth factor (VEGF) can promote vascular endothelial cells proliferation, increase vascular permeability, and regulate thrombus formation.^8^, ^9^ Past pre‐clinical studies demonstrated that serum VEGF levels were significantly elevated in CAD and IS.^10^, ^11^ Moreover, VEGF was also shown to be able to cause growth of atherosclerotic lesions or even plaque rupture in animal studies.^12^, ^13^ VEGF receptor 2 (*VEGFR2*) is the principal receptor of VEGF in blood vessels.^14^ Consequently, it is possible that functional *VEGFR2* polymorphisms, which could influence the normal biological function of VEGF, may also affect individual susceptibility to ASCVD.

In recent years, some investigations already studied potential associations between *VEGFR2* polymorphisms and ASCVD. Nevertheless, the findings of these studies were not always consistent and the sample size of each study was also statistically insufficient. In this meta‐analysis, we aimed to better analyze the relationship between *VEGFR2* polymorphisms and ASCVD in a larger combined population.

## 2 MATERIALS AND METHODS

This meta‐analysis was written in accordance with Preferred Reporting Items for Systematic Reviews and Meta‐analyses (PRISMA) checklist.^15^ We created an Open Science Framework (osf.io) account to make this meta‐analysis more publicly available.

### 2.1 Literature search and inclusion criteria

Eligible articles published before May 2019 were retrieved from PubMed, Web of Science, Embase, and CNKI by using the following key words: (“Vascular Endothelial Growth Factor Receptor‐2” OR “VEGFR‐2” OR “Vascular Endothelial Growth Factor Receptor 2” OR “VEGFR2” OR “Kinase Insert Domain Receptor” OR “KDR”) AND (“polymorphism” OR “variant” OR “variation” OR “SNP” OR “mutation” OR “genome‐wide association study” OR “genetic association study”) AND (“atherosclerosis” OR “arteriosclerosis” OR “coronary heart disease” OR “coronary artery disease” OR “angina pectoris” OR “acute coronary syndrome” OR “myocardial infarction” OR “ischemic stroke” OR “cerebral infarction” OR “brain infarction” OR “transient ischemic attack” OR “peripheral arterial disease”). Additionally, we also checked the reference lists of all retrieved articles.

Inclusion criteria for this meta‐analysis were as follows: (a) genetic association study about *VEGFR2* polymorphisms and ASCVD in human beings; (b) providing distributions of genotypes in cases and controls; (c) available full text in English or native language of the authors (Chinese). We excluded studies when more than one of the following conditions was met: (a) studies that were not about *VEGFR2* polymorphisms and ASCVD; (b) reviews or comments; (c) case reports or case series. If we found repeated publications by the same authors, only the most comprehensive study was included for this meta‐analysis.

### 2.2 Data extraction and quality assessment

Following information was extracted by two authors: the last name of the first author and publication year, country of the principal investigator and ethnicity of study participants, type of disease, total sample size of each study and the distribution of *VEGFR2* polymorphisms in cases and controls. We also calculated the probability value (*P*‐value) of Hardy–Weinberg equilibrium (HWE).

Newcastle–Ottawa scale (NOS) was used to evaluate the methodology quality of eligible studies.^16^ The score of this scale ranged between zero and nine, if a study scored seven or more, we thought that the quality of this study was acceptable.

Data extraction and quality assessment were conducted by two authors independently. We wrote to the corresponding authors for extra information when we thought that important information was missed.

### 2.3 Statistical analyses

Review Manager Version 5.3.3 was used in this meta‐analysis for statistical analyses. We used the *Z* test to assess whether *VEGFR2* polymorphisms were significantly associated with ASCVD, with the statistical significance *P* level set at .05. *I* ^2^ statistics were used to evaluate between‐study heterogeneities. Random‐effect models (DerSimonian‐Laird method) were used if *I* ^2^ exceeded 50%. Otherwise, meta‐analyses were conducted with fixed‐effect models (Mantel–Haenszel method). We also conducted subgroup analyses by type of disease. We tested the robustness of synthetic results in sensitivity analyses. We evaluated publication biases by funnel plots.

## 3 RESULTS

### 3.1 Characteristics of included studies

**Figure 1:**
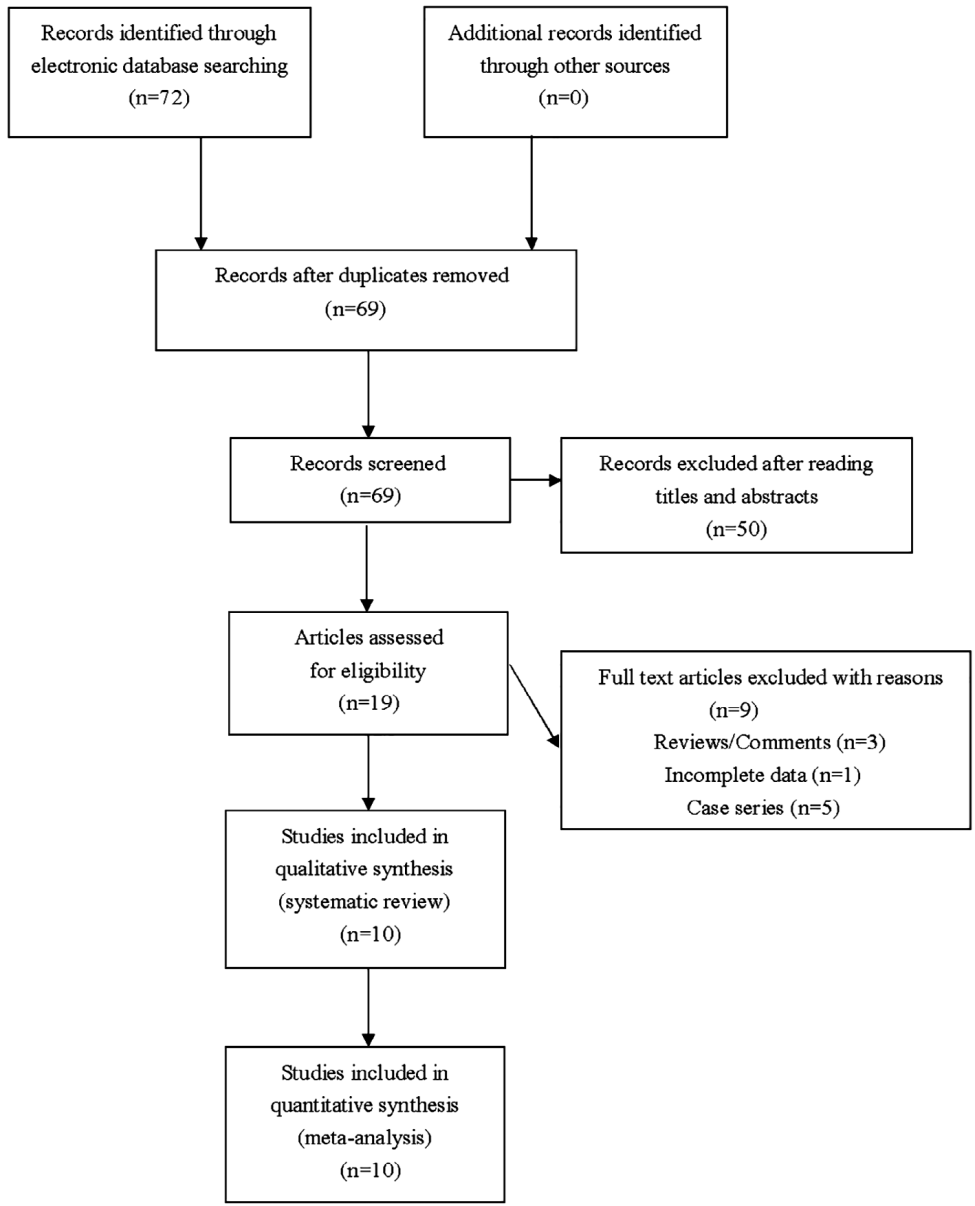
Flowchart of study selection for the present study

Seventy‐two articles were identified by our comprehensive literature searching. Nineteen articles were retrieved for eligibility assessment after exclusion of irrelevant and duplicate articles. Another three reviews and five case series were subsequently excluded, and one study was excluded due to lack of essential data. Totally 10 eligible studies were ultimately included for this meta‐analysis (Figure 1). Table 1 presented essential data extracted from included studies.

**Table 1:** The characteristics of included studies

First author, year	Country	Ethnicity	Type of disease	Sample size	Genotype distribution		*P*‐value for HWE	NOS score
					Cases	Controls		
**rs1870377**					AA/AT/TT			
Han, 2012^24^	Korea	Asian	IS	383/387	113/188/82	129/185/73	.644	8
Li, 2016^26^	China	Asian	CAD	533/533	311/183/39	325/192/16	.049	8
Liu, 2016^27^	China	Asian	CAD	810/805	261/206/343	254/291/260	<.001	8
Oh, 2011^29^	Korea	Asian	IS	501/478	119/262/120	159/236/83	.775	8
Wang, 2007^31^	China	Asian	CAD	1034/1640	278/543/213	560/795/285	.921	8
Xin, 2014^32^	China	Asian	CAD	200/200	58/100/42	68/98/34	.897	7
**rs2071559**					TT/TC/CC			
Han, 2012^24^	Korea	Asian	IS	383/387	202/151/30	229/133/25	.343	8
Kariž, 2014^25^	Slovenia	Caucasian	MI	171/850	36/88/47	236/431/183	.598	8
Li, 2016^26^	China	Asian	CAD	533/533	122/253/158	143/261/129	.645	8
Merlo, 2016^28^	Slovenia	Caucasian	Atherosclerosis	500/95	133/250/117	22/59/14	.014	7
Oh, 2011^29^	Korea	Asian	IS	501/478	236/224/41	241/196/41	.898	8
Shen, 2014^30^	China	Asian	IS	43/103	14/19/10	86/13/4	.002	8
Wang, 2007^31^	China	Asian	CAD	1034/1640	441/462/131	806/657/177	.014	8
Xin, 2014^32^	China	Asian	CAD	200/200	84/89/27	97/80/23	.301	7
Zhang, 2007^33^	China	Asian	IS	530/1798	247/227/56	862/751/185	.259	8
**rs2305948**					GG/GA/AA			
Han, 2012^24^	Korea	Asian	IS	383/387	307/71/5	299/83/5	.778	8
Kariž, 2014^25^	Slovenia	Caucasian	MI	171/850	26/75/70	148/387/315	.123	8
Li, 2016^26^	China	Asian	CAD	533/533	388/122/23	416/105/12	.088	8
Liu, 2016^27^	China	Asian	CAD	810/805	151/207/452	205/309/291	<.001	8
Oh, 2011^29^	Korea	Asian	IS	501/478	381/112/8	378/95/5	.720	8
Wang, 2007^31^	China	Asian	CAD	1034/1640	800//221/13	1362/261/17	.260	8
Xin, 2014^32^	China	Asian	CAD	200/200	158/40/2	164/34/2	.873	7
Zhang, 2009^33^	China	Asian	IS	812/1798	620/182/10	1429/351/18	.488	8

*Note*: HWE assumes that allele and genotype frequencies in a population will remain constant from generation to generation in the absence of other evolutionary influences. Consider a population of monoecious diploids, where each organism produces male and female gametes at equal frequency, and has two alleles at each gene locus. The allele frequencies at each generation are obtained by pooling together the alleles from each genotype of the same generation according to the expected contribution from the homozygote and heterozygote genotypes.

Abbreviations: CAD, coronary artery disease; MI, myocardial infarction; IS, ischemic stroke; HWE, Hardy–Weinberg equilibrium; NOS, Newcastle–Ottawa scale.

### 3.2 Meta‐analyses results

The *VEGFR2* rs1870377 (dominant comparison: 0.81 (0.73‐0.89), *I* ^2^ = 56%; recessive comparison: 1.40 (1.25‐1.57), *I* ^2^ = 34%; allele comparison: 0.81 (0.76‐0.87), *I* ^2^ = 0%), rs2071559 (dominant comparison: 0.83 (0.76‐0.91), *I* ^2^ = 0%; recessive comparison: 1.22 (1.07‐1.38), *I* ^2^ = 0%; allele comparison: 0.86 (0.81‐0.92), *I* ^2^ = 0%) and rs2305948 (dominant comparison: 0.79 (0.72‐0.87), *I* ^2^ = 25%; recessive comparison: 1.44 (1.08‐1.92), *I* ^2^ = 60%; allele comparison: 0.79 (0.68‐0.92), *I* ^2^ = 73%) polymorphisms were all found to be significantly associated with susceptibility to ASCVD in general population. Subgroup analyses by type of disease revealed similar significant findings for rs1870377, rs2071559, and rs2305948 polymorphisms in CAD subgroup. Moreover, positive results were also found for rs1870377 polymorphism in IS subgroup (Table 2).

**Table 2:** Results of overall and subgroup analyses

Polymorphisms	Population	Sample size	Dominant comparison			Recessive comparison			Over‐dominant comparison			Allele comparison		
			*P*‐value	OR (95%CI)	*I* ^2^	*P*‐value	OR (95%CI)	*I* ^2^	*P‐*value	OR (95%CI)	*I* ^2^	*P*‐value	OR (95%CI)	*I* ^2^
**rs1870377**	Overall	3461/4043	**<.0001**	**0.81 (0.73‐0.89)**	56%	**<.0001**	**1.40 (1.25‐1.57)**	34%	.74	0.96 (0.77‐1.21)	81%	**<.0001**	**0.81 (0.76‐0.87)**	0%
	Asian	3461/4043	**<.0001**	**0.81 (0.73‐0.89)**	56%	**<.0001**	**1.40 (1.25‐1.57)**	34%	.74	0.96 (0.77‐1.21)	81%	**<.0001**	**0.81 (0.76‐0.87)**	0%
	CAD	2577/3178	.10	0.85 (0.70‐1.03)	61%	**.001**	**1.46 (1.17‐1.84)**	53%	.57	0.91 (0.65‐1.27)	88%	**<.0001**	**0.82 (0.76‐0.88)**	0%
	IS	884/865	**.001**	**0.71 (0.58‐0.88)**	48%	**.01**	**1.35 (1.06‐1.70)**	4%	.36	1.09 (0.91‐1.32)	0%	**.0006**	**0.79 (0.69‐0.90)**	0%
**rs2071559**	Overall	3856/5981	**<.0001**	**0.83 (0.76‐0.91)**	0%	**.002**	**1.22 (1.07‐1.38)**	0%	.05	1.09 (1.00‐1.19)	36%	**<.0001**	**0.86 (0.81‐0.92)**	0%
	Asian	3181/5036	**<.0001**	**0.83 (0.75‐0.91)**	0%	**.03**	**1.17 (1.02‐1.34)**	0%	**.01**	**1.12 (1.02‐1.23)**	0%	**<.0001**	**0.87 (0.81‐0.93)**	0%
	Caucasian	671/945	.67	0.89 (0.52‐1.52)	63%	**.01**	**1.49 (1.09‐2.05)**	0%	.42	0.81 (0.49‐1.35)	71%	**.04**	**0.82 (0.68‐0.99)**	0%
	CAD	1938/3223	**<.0001**	**0.77 (0.68‐0.87)**	0%	**.003**	**1.27 (1.08‐1.48)**	0%	.07	1.12 (0.99‐1.25)	8%	**<.0001**	**0.82 (0.75‐0.89)**	0%
	IS	1414/2663	.07	0.88 (0.77‐1.01)	0%	.74	1.04 (0.82‐1.32)	0%	.11	1.12 (0.98‐1.28)	0%	.12	0.92 (0.83‐1.02)	0%
**rs2305948**	Overall	4444/6691	**<.0001**	**0.79 (0.72‐0.87)**	25%	**.01**	**1.44 (1.08‐1.92)**	60%	.97	1.00 (0.78‐1.29)	86%	**.002**	**0.79 (0.68‐0.92)**	73%
	Asian	4273/5841	**<.0001**	**0.79 (0.72‐0.87)**	35%	**.009**	**1.52 (1.11‐2.08)**	53%	.99	1.00 (0.76‐1.33)	88%	**.008**	**0.80 (0.67‐0.94)**	77%
	CAD	2748/4028	**<.0001**	**0.73 (0.64‐0.82)**	0%	**.03**	**1.47 (1.03‐2.10)**	75%	.91	0.98 (0.66‐1.44)	91%	**.0002**	**0.72 (0.61‐0.86)**	71%
	IS	1696/2663	.14	0.89 (0.77‐1.04)	38%	.44	1.25 (0.71‐2.21)	0%	.19	1.11 (0.95‐1.29)	35%	.12	0.90 (0.78‐1.03)	34%

*Note*: The values in bold represent there is statistically significant differences between cases and controls.

Abbreviations: CAD, coronary artery disease; CI, Confidence interval; IS, ischemic stroke; OR, odds ratio.

### 3.3 Sensitivity analyses

We tested the effects of each study on meta‐analysis results in sensitivity analyses. The meta‐analysis results remained unchanged in sensitivity analyses, suggesting that our findings were statistically robust.

### 3.4 Publication biases

We evaluated publication biases by using funnel plots. We did not observe dissymmetry in any funnel plots, which indicated that the possibility that our meta‐analysis results were affected by overt publication biases was low (Figure S1).

## 4 DISCUSSION

In this meta‐analysis, the combined results revealed that *VEGFR2* rs1870377, rs2071559, and rs2305948 polymorphisms were all significantly associated with CAD. Moreover, rs1870377 polymorphism was also found to be significantly associated with IS. The meta‐analysis results remained unchanged in sensitivity analyses, suggesting that our combined results were statistically robust.

There are few points that should be considered when interpreting our meta‐analysis results. First, pre‐clinical studies proved that the minor allele of rs2071559 polymorphism (−604 T > C) could lead to decreased transcription activity of *VEGFR2*, whereas the minor alleles of rs1870377 (+1719A > T) and rs2305948 (+1192G > A) polymorphisms were associated with reduced binding affinity of *VEGFR2*.^17^ So theoretically, it is possible that these three functional genetic variations may impact biological function of *VEGFR2* and VEGF, and ultimately influence individual susceptibility to ASCVD. Second, in stratified analyses, we noticed that the positive results were mainly driven by the CAD subgroup, which suggested that the magnitude of effects of *VEGFR2* polymorphisms on individual susceptibility to CAD and IS might be somewhat different. However, given that the trends of associations in CAD and IS were similar, and the sample sizes of combined analyses with regard to IS were still relatively small. Maybe our meta‐analysis was still not statistically sufficient to detect the actual relationship between *VEGFR2* polymorphisms and IS. So we call on further genetic association studies to confirm our findings, especially for *VEGFR2* polymorphisms and IS. Third, the etiology of ASCVD is very complicated, consequently, we strongly recommend future studies to conduct haplotype analyses and investigate potential gene‐gene interactions to more comprehensively explore the effects of genetics on disease susceptibility.^18^ Fourth, most eligible studies were from Asian countries, but studies of Caucasian and African countries are still scarce.

This meta‐analysis has some limitations. First, our meta‐analysis results were derived from unadjusted combined analyses, and failure to adjust for some crucial variables may impact the precision of our findings.^19^, ^20^ Second, environmental factors may also affect the relationship between *VEGFR2* polymorphisms and ASCVD. Regrettably, most of included studies only focus on genetic associations, so we could not conduct analyses regarding genetic‐environmental interactions.^21^, ^22^ Third, we did not search for gray literatures. So although we did not observe dissymmetry in any funnel plots, there is still possibility that publication biases may influence our meta‐analysis results.^23^ Fourth, during literature searching, we did not find sufficient literatures to support combined analyses for other *VEGFR2* polymorphisms. Since no any other *VEGFR2* polymorphisms were studied by at least two eligible studies with regard to their associations with ASCVD, this meta‐analysis only focus on relationship between three common *VEGFR2* (rs1870377, rs2071559, and rs2305948) polymorphisms and ASCVD.

## 5 CONCLUSIONS

In summary, this meta‐analysis proved that *VEGFR2* rs1870377, rs2071559, and rs2305948 polymorphisms could be used to identify individual with elevated susceptibility to CAD. Moreover, rs1870377 polymorphism could be used to identify individual with elevated susceptibility to IS. However, further studies with larger sample sizes still need to verify our findings, especially for IS.

## CONFLICT OF INTEREST

The authors declare no potential conflict of interests.

## AUTHOR CONTRIBUTIONS

Li Wang and Bing Wang conceived and designed this study. Li Wang and Hui Ge conducted the systematic literature review. Longyun Peng performed data analyses. Li Wang and Bing Wang drafted the manuscript. All authors gave final approval and agree to be accountable for all aspects of work ensuring integrity and accuracy.

## ETHICS STATEMENT

This article does not contain any studies with human participants or animals performed by any of the authors, thus no ethical approval is required.

## Supporting information


**FIGURE S1** Funnel plots
