# Supplementary material for: A meta‐analysis of the relationship between VEGFR2 polymorphisms and atherosclerotic cardiovascular diseases
Source: Clin Cardiol. 2019 Jul 24;42(10):860–5. doi: 10.1002/clc.23233 (PMC6788482; doi:10.1002/clc.23233)
Supplement: Supplementary file 1 — FIGURE S1 Funnel plots [file CLC-42-860-s001.docx]

**Supplementary figure 1. Funnel plots**


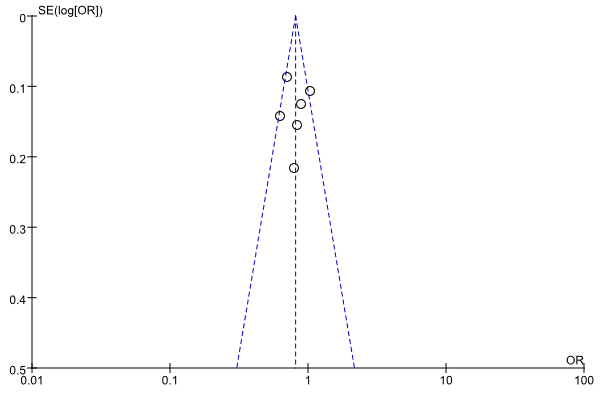


Funnel plot of rs1870377 polymorphism and ASCVD under dominant comparison


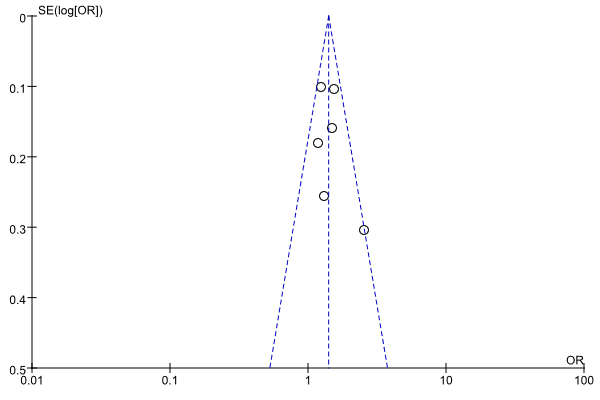


Funnel plot of rs1870377 polymorphism and ASCVD under recessive comparison


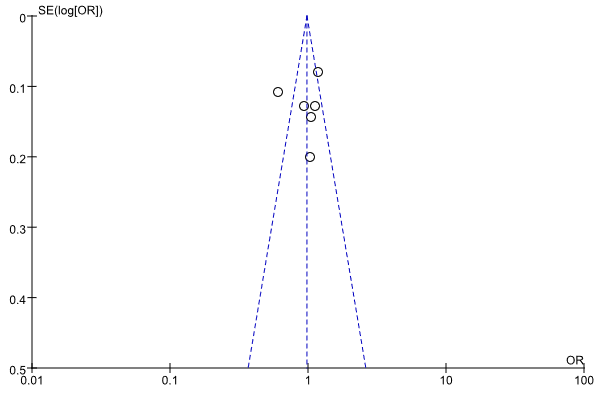


Funnel plot of rs1870377 polymorphism and ASCVD under over-dominant comparison


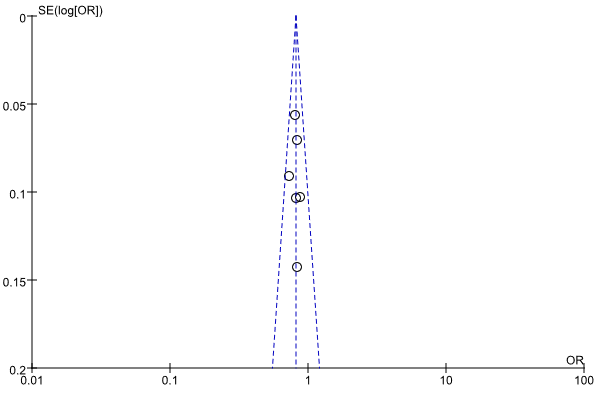


Funnel plot of rs1870377 polymorphism and ASCVD under allele comparison


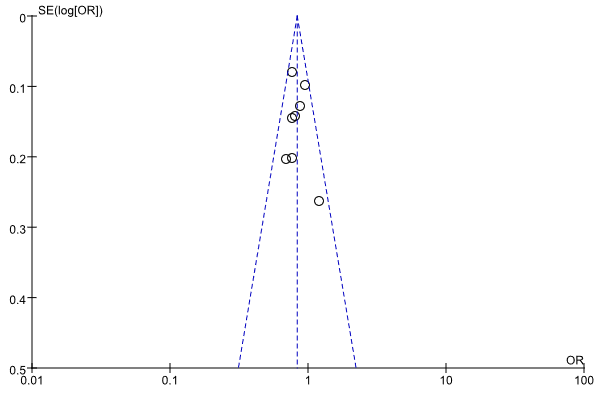


Funnel plot of rs2071559 polymorphism and ASCVD under dominant comparison


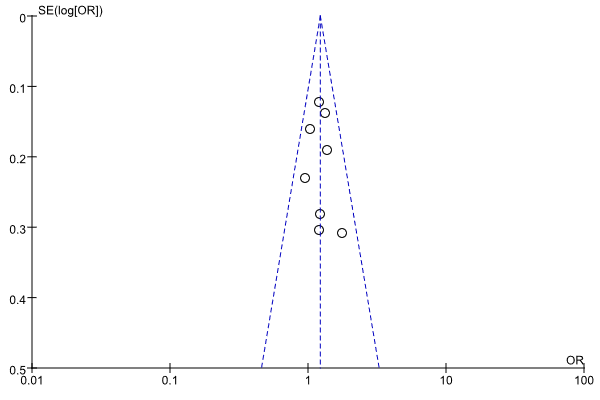


Funnel plot of rs2071559 polymorphism and ASCVD under recessive comparison


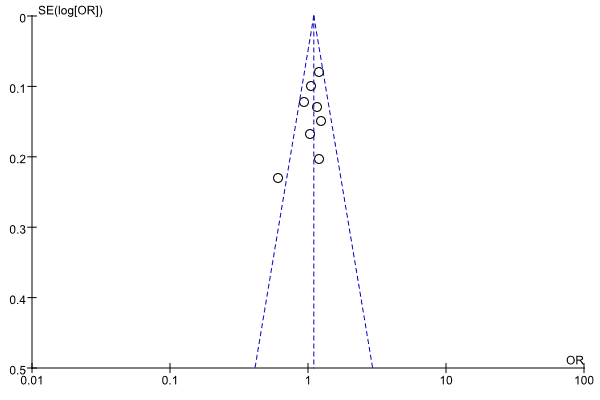


Funnel plot of rs2071559 polymorphism and ASCVD under over-dominant comparison


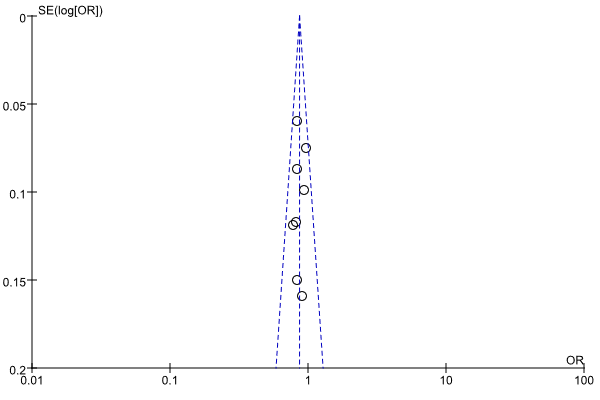


Funnel plot of rs2071559 polymorphism and ASCVD under allele comparison


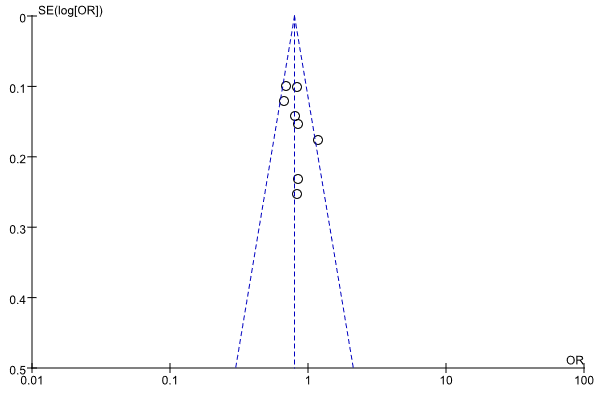


Funnel plot of rs2305948 polymorphism and ASCVD under dominant comparison


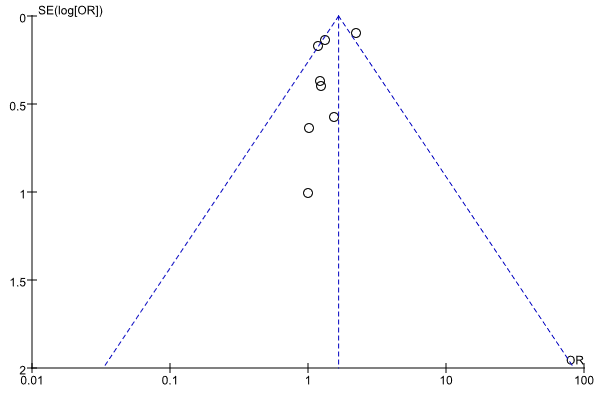


Funnel plot of rs2305948 polymorphism and ASCVD under recessive comparison


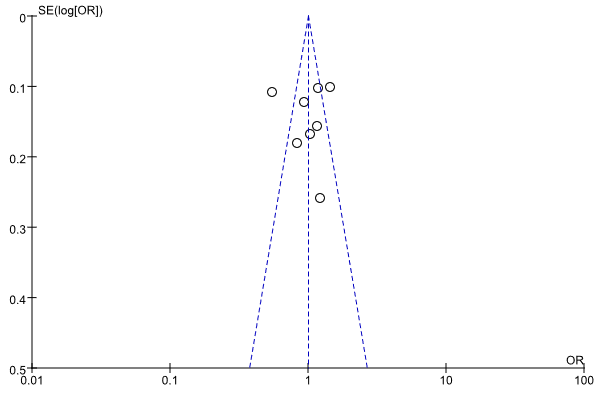


Funnel plot of rs2305948 polymorphism and ASCVD under over-dominant comparison


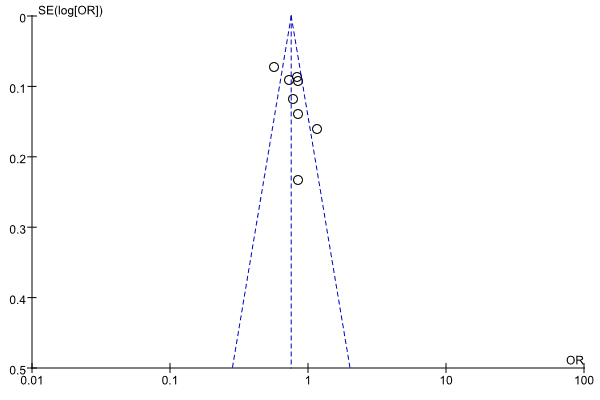


Funnel plot of rs2305948 polymorphism and ASCVD under allele comparison
